# Mean Platelet Volume and Mean Platelet Volume-to-Platelet Ratio and Their Association With Mortality in Patients Undergoing Maintenance Hemodialysis

**DOI:** 10.7759/cureus.114527

**Published:** 2026-08-14

**Authors:** Diana D Nenova, Yanko G Yankov, Gergana M Chausheva

**Affiliations:** 1 Department of Nephrology, Medical University "Prof. Dr. Paraskev Stoyanov", Varna, BGR; 2 Clinic of Nephrology and Dialysis, University Hospital "St. Marina", Varna, BGR; 3 Clinic of Maxillofacial Surgery, University Hospital "St. Marina", Varna, BGR; 4 Department of General and Operative Surgery, Medical University "Prof. Dr. Paraskev Stoyanov", Varna, BGR; 5 Department of Clinical Laboratory, Medical University "Prof. Dr. Paraskev Stoyanov", Varna, BGR; 6 Department of Central Clinical Laboratory, University Hospital "St. Marina", Varna, BGR

**Keywords:** cardiovascular risk, chronic kidney disease, death, dialysis, mean platelet volume, mean platelet volume-to-platelet ratio, platelet activation, prognosis, risk stratification, survival

## Abstract

Introduction

Patients receiving maintenance hemodialysis (HD) remain at high risk of cardiovascular morbidity and mortality despite advances in dialysis therapy. The mean platelet volume (MPV) reflects platelet activation and has emerged as a potential prognostic biomarker, while the mean platelet volume-to-platelet ratio (MPR) may provide additional information regarding thrombo-inflammatory status. This study evaluated the associations of MPV and MPR with long-term survival, cardiovascular mortality, dialysis adequacy, and anemia-related parameters in maintenance HD patients.

Materials and methods

This retrospective single-center observational study included 98 adult patients undergoing maintenance HD at the Department of Nephrology and Dialysis, University Hospital "St. Marina", Varna, Bulgaria. Patients were stratified according to baseline MPV into three groups: <10.0 fL (n=38), 10.0-11.0 fL (n=32), and >11.0 fL (n=28). Clinical, laboratory, and dialysis-related parameters were analyzed using annual mean values. Long-term survival was analyzed using Kaplan-Meier estimates, and survival distributions were compared with the log-rank test. The associations of MPV and MPR with all-cause mortality were subsequently examined using univariable Cox proportional hazards regression, with effect estimates reported as hazard ratios and corresponding 95% CI.

Results

Baseline demographic and clinical characteristics were comparable among the three groups. Increasing MPV was associated with progressively lower platelet counts, reduced dialysis adequacy (URR), lower hemoglobin and serum albumin concentrations, and higher erythropoiesis-stimulating agent (ESA) requirements (all p<0.001). MPV correlated positively with weekly ESA dose (r=0.644; p=0.002). Five-year survival declined from 86.8% in the lowest MPV group to 71.9% and 46.4% in the intermediate and high MPV groups, respectively (log-rank χ²=12.69; p=0.0018). Patients with MPV >11.0 fL had a significantly higher risk of all-cause mortality than those with MPV <10.0 fL (HR 5.45; 95% CI 1.98-15.02; p=0.001). In univariable Cox regression, patients with MPV >11.0 fL had a significantly higher hazard of all-cause mortality than those with MPV <10.0 fL (HR 5.45; 95% CI 1.98-15.02; p=0.001). Each 0.01-unit increase in MPR was associated with a 49% higher hazard of death (HR 1.49; 95% CI 1.15-2.28; p<0.001). Cardiovascular mortality also increased progressively with higher MPV values.

Discussion

The findings suggest that increased platelet activation, reflected by elevated MPV and MPR, is associated with adverse clinical outcomes in maintenance HD patients. MPR showed a strong association with all-cause mortality and may provide complementary prognostic information to MPV alone. These results are consistent with the concept that platelet activation contributes to the chronic inflammatory and thrombotic milieu characteristic of end-stage kidney disease. However, given the retrospective observational design and lack of multivariable adjustment, these findings should be interpreted as hypothesis-generating.

Conclusions

Elevated MPV and MPR were associated with impaired dialysis adequacy, greater ESA requirements, lower hemoglobin and albumin concentrations, increased cardiovascular mortality, and reduced long-term survival. As inexpensive and routinely available hematological indices, MPV and MPR may have potential value for risk stratification in maintenance HD patients.

## Introduction

Risk stratification in maintenance hemodialysis (HD) remains challenging because conventional cardiovascular risk (CVR) factors incompletely explain the high mortality observed in this population [1]. Although dialysis technology and the management of renal anemia have improved markedly over recent decades, cardiovascular disease remains the leading cause of death in patients with end-stage renal disease (ESRD), with mortality rates far exceeding those observed in the general population. Traditional CVR factors alone do not fully explain this excess risk, highlighting the need for additional biomarkers that reflect the complex interplay of inflammation, oxidative stress, endothelial dysfunction, platelet activation, and accelerated atherosclerosis characteristic of ESRD [2-4].

Abnormal platelet activation and function are thought to play a central role in the pathogenesis of cardiovascular disease in individuals with chronic kidney disease. In addition to impaired platelet aggregation and adhesion, enhanced platelet activation contributes to thrombo-inflammatory processes that promote vascular injury, vascular access dysfunction, and cardiovascular events [5,6]. The mean platelet volume (MPV), routinely reported as part of the complete blood count, reflects the average size of circulating platelets and is widely recognized as an indirect surrogate of platelet activation [7]. Larger platelets are metabolically and enzymatically more active, possess greater prothrombotic potential, and produce higher amounts of thromboxane A₂ and pro-inflammatory mediators than smaller platelets [7,8].

Previous studies have identified elevated MPV as a marker of adverse cardiovascular outcomes in the general population and in several high-risk patient groups, including those with diabetes mellitus, coronary artery disease, and chronic kidney disease [9-11]. In contrast, the evidence supporting its prognostic utility in maintenance HD remains inconclusive. While some investigators have reported associations between increased MPV and mortality, vascular access thrombosis, and cardiovascular events, others have failed to demonstrate independent predictive value [12]. These discrepancies may reflect differences in study design, patient characteristics, laboratory methodology, and dialysis protocols.

Recently, the mean platelet volume-to-platelet ratio (MPR) has emerged as a potentially informative biomarker that incorporates information from both MPV and platelet count. By integrating platelet size and platelet count into a single index, MPR may provide additional information regarding platelet activation and the underlying inflammatory and thrombotic state. Although MPR has shown prognostic significance in several cardiovascular and inflammatory conditions, its clinical relevance in maintenance HD has received limited investigation [13].

In addition to platelet activation, dialysis adequacy, anemia, and nutritional status are well-established determinants of clinical outcomes in HD patients. Examining the relationships between MPV, MPR, dialysis adequacy, erythropoiesis-stimulating agent (ESA) requirements, hemoglobin (Hgb) concentration, and serum albumin may therefore provide further insight into the mechanisms linking platelet activation with adverse prognosis.

The aim of the present study was to evaluate the associations of MPV and MPR with all-cause and cardiovascular mortality in patients treated with maintenance HD. Secondary objectives were to investigate the relationships between platelet indices, dialysis adequacy, and hematological parameters and to explore the potential prognostic relevance of MPV and MPR as readily available biomarkers in this high-risk population.

## Materials and methods

Study design and patient population

A retrospective observational study was conducted at the Department of Nephrology and Dialysis, University Hospital "St. Marina", Varna, Bulgaria, over a five-year period (January 2018 to December 2023). The study included 98 adult patients receiving maintenance HD.

Patients were stratified into three groups according to their baseline MPV values: Group 1 with MPV <10.0 fL (n=38), Group 2 with MPV between 10.0 and 11.0 fL (n=32), and Group 3 with MPV >11.0 fL (n=28).

Participants and eligibility criteria

A total of 147 patients receiving maintenance HD were initially screened for study participation. Eligible participants were adults (≥18 years) who had received maintenance HD for at least six months and had minimal residual kidney function, defined as a daily urine output of less than 100 mL. Additional eligibility criteria included treatment with a standardized baseline dose of short-acting ESA (47±3.1 IU/kg), anticoagulation with low-molecular-weight heparin, and a uniform antiplatelet regimen consisting of aspirin at an identical daily dose. Complete clinical and laboratory data were also required for study enrollment.

Patients were not eligible for inclusion if they were younger than 18 years, had received HD for less than six months, or were dialyzed through a temporary vascular access. Individuals with marked differences in their initial ESA dose were also excluded to minimize variability in anemia management and its potential influence on platelet-related parameters. Additional exclusion criteria comprised active malignancy, hematological disorders, untreated iron deficiency, ongoing bleeding, corticosteroid or immunosuppressive therapy, recent blood transfusion or administration of granulocyte colony-stimulating factors, clinically or laboratory-confirmed active infection, treatment with unfractionated heparin because of the potential risk of heparin-induced thrombocytopenia, and incomplete medical records.

Of the 147 patients initially screened, 14 were excluded because of incomplete documentation, leaving 133 eligible patients. Application of the predefined exclusion criteria resulted in the exclusion of an additional 35 patients, including seven with temporary vascular access, six with non-standardized baseline ESA dosing, seven with malignant disease, five who had recently received blood transfusions, and 10 receiving corticosteroid therapy. Following patient screening and application of the predefined eligibility criteria, the study population comprised 98 maintenance HD patients. Thus, of the 147 patients initially screened, 49 were excluded, and 98 were included in the final analysis.

Excluded patients had a mean age of 54.3±3.6 years, which was comparable to that of the included cohort. Moreover, consecutive enrollment of all patients meeting the predefined eligibility criteria throughout the five-year study period was intended to minimize selection bias and improve the representativeness of the study sample. Eligibility and exclusion criteria were established before data collection and were based exclusively on objective clinical parameters, thereby minimizing the risk of investigator-related selection bias. Furthermore, the dataset was complete for all primary study variables, eliminating the need for data imputation and reducing the potential for bias associated with missing information.

HD protocol

All patients underwent conventional bicarbonate HD using Fresenius Medical Care 5008 and 4008 dialysis systems (Fresenius Medical Care AG & Co., Bad Homburg v. d. Höhe, Germany). Dialysis was performed three times weekly, with each session lasting four hours.

High-flux dialyzers with membrane surface areas (1.8-2.0 m^2^) individually selected according to each patient's body surface area were used throughout the study. Uniform dialysis prescriptions were applied throughout the study, with treatment delivered at a blood flow rate (Qb) of 280 mL/min and a dialysate flow rate (Qd) of 500 mL/min.

All patients received anticoagulation with low-molecular-weight heparin (Fraxiparine®, Aspen Pharma Trading Limited, Dublin, Ireland) administered according to body weight.

Laboratory measurements

All laboratory analyses were performed as part of routine patient care in the Central Clinical Laboratory at the University Hospital "St. Marina", Varna, Bulgaria, following national quality assurance standards. Blood samples were obtained using the standardized stop-pump technique, with collection performed immediately before and, where appropriate, after the HD session to minimize the effects of vascular access recirculation and post-dialysis urea rebound.

Hgb concentration (g/L) was measured using the sodium lauryl sulfate colorimetric method on an automated six-part differential hematology analyzer (Sysmex XN-1000, Siemens Healthcare, Forchheim, Germany). The laboratory reference interval for Hgb was 120-180 g/L, while the therapeutic target for patients with end-stage kidney disease receiving maintenance HD was 110-120 g/L.

Platelet count (×10⁹/L) was determined by the electrical impedance method, whereas MPV (fL) was generated automatically by the analyzer based on the ratio of plateletcrit to platelet count. The laboratory reference ranges were 140-440×10⁹/L for platelets and 6.0-10.0 fL for MPV. MPR was derived for each patient by dividing MPV by the corresponding platelet count. Complete blood count samples were collected into ethylenediaminetetraacetic acid (EDTA)-anticoagulated tubes and analyzed within 40 minutes after venipuncture, without freezing or prolonged storage, in accordance with the laboratory's standard operating procedures.

Serum urea (mmol/L) was quantified using a UV kinetic assay based on the glutamate dehydrogenase (GLDH) enzymatic reaction on an ADVIA Chemistry 1800 analyzer (Siemens Healthcare, Forchheim, Germany). The reference interval for serum urea was 3.2-8.2 mmol/L. Creatinine and albumin were analyzed on the same platform using the kinetic Jaffé and bromocresol green colorimetric methods, respectively. The corresponding laboratory reference intervals were 44-115 μmol/L for creatinine and 32-48 g/L for albumin.

To minimize the impact of ultrafiltration-related hemoconcentration, hematological analyses, including MPV, platelet, and Hgb, were performed exclusively on pre-dialysis blood samples. Serum urea and creatinine concentrations, however, were determined before and after each dialysis session to assess treatment adequacy, and these values were used to calculate the urea reduction ratio (URR).

URR was derived from pre- and post-dialysis serum urea concentrations using the formula $\text{URR (\%)}=100\times(\mathrm{1&minus;C/Co})$, where Co and C denote the pre- and post-dialysis values, respectively.

Data collection

To improve the reliability of the analyzed variables and reduce the influence of short-term biological fluctuations, statistical analyses were based on annual mean values rather than individual laboratory measurements. Clinical, laboratory, and dialysis-related parameters were extracted retrospectively from routinely collected medical records throughout the study period.

At our dialysis center, hematological indices and dialysis adequacy parameters are monitored approximately six times per year for each patient as part of standard clinical follow-up. For every participant, the arithmetic mean of these repeated measurements was calculated and used as the representative value for subsequent analyses. This approach provided a more robust estimate of each patient's long-term clinical status while minimizing the impact of transient physiological variation. The use of annual mean values was chosen a priori to reduce within-patient variability related to short-term biological and dialysis-related fluctuations and to provide a standardized summary measure based on repeated observations for each participant. Consequently, repeated longitudinal observations were condensed into a single representative value per patient for cross-sectional statistical comparisons.

Bias minimization

Several measures were implemented to minimize potential sources of bias. Treatment-related variability was reduced by ensuring that all patients received standardized ESA therapy, low-molecular-weight heparin anticoagulation, and a uniform aspirin-based antiplatelet regimen. To reduce the influence of potential confounding factors known to influence platelet morphology and function, patients with hematological disorders, active infection, malignancy, recent blood transfusion, corticosteroid or immunosuppressive therapy, uncorrected iron deficiency, or active bleeding were excluded.

Methodological bias was further minimized through standardized collection of pre-dialysis blood samples for hematological analyses and the use of uniform dialysis equipment and treatment protocols throughout the study period. Selection bias was minimized through the consecutive inclusion of all patients who satisfied the predefined eligibility criteria throughout the study period. Complete clinical and laboratory data were available for all variables included in the analyses; therefore, no imputation procedures were necessary, and the risk of bias arising from missing data was minimized.

Despite these measures, the retrospective observational design precludes complete elimination of residual confounding, and the influence of unmeasured variables cannot be entirely excluded. In addition, the survival analyses were not adjusted for established mortality risk factors in a multivariable Cox model; therefore, the reported hazard ratios (HRs) should be interpreted as unadjusted associations rather than independent prognostic effects.

Statistical analysis

Statistical analyses were conducted using IBM SPSS Statistics for Windows, Version 20.0 (IBM Corp., Armonk, New York, United States). Continuous variables are presented as mean±standard deviation (SD), whereas categorical variables are summarized as counts and percentages.

The distribution of continuous variables was assessed by the Shapiro-Wilk test together with visual inspection of histogram plots. Depending on the distribution and the number of groups compared, continuous variables were analyzed using either one-way analysis of variance (ANOVA) or the independent-samples Student's t-test. Associations between categorical variables were evaluated using Pearson's chi-squared (χ²) test.

Linear relationships between continuous variables were examined using Pearson's correlation coefficient (r). Survival probabilities were estimated using the Kaplan-Meier method, and differences between survival curves were evaluated with the log-rank test. The associations of baseline MPV group and the MPR with all-cause mortality were assessed using univariable Cox proportional hazards regression, with results reported as HRs and 95% CI. For MPR, the HR was expressed per 0.01-unit increase. No multivariable adjustment for potential confounders was performed; therefore, the Cox regression estimates represent unadjusted associations and should not be interpreted as independent prognostic effects. Relative risks (RRs) with corresponding 95% CI were additionally calculated to compare all-cause and cardiovascular mortality across the three MPV groups.

Because this was a retrospective analysis of an existing cohort, no a priori sample size calculation was performed. The study included all patients who met the predefined eligibility criteria during the study period.

All statistical analyses were two-sided, and statistical significance was defined as a p-value of <0.05.

Ethical considerations

The Commission on Ethics of Research at the Medical University "Prof. Dr. Paraskev Stoyanov" approved the study protocol (approval number: 107/28.10.2021). Owing to the retrospective nature of the study and the use of fully anonymized data obtained from routine clinical practice, the requirement for written informed consent was waived.

A proportion of the patients included in this cohort were previously enrolled in the doctoral dissertation entitled "Adequacy of Dialysis Treatment and the Relationship With Achieved Quality of Life and Survival in Patients With Stage V Chronic Kidney Disease" by Diana Nenova, defended in April 2022 [2]. In addition, a subset of the cohort contributed to previously published studies addressing different research questions. The present study constitutes a secondary analysis of the cohort with a distinct scientific objective, specifically evaluating the association of MPV and MPR with long-term survival and cardiovascular mortality in maintenance HD patients. The analyses presented here, including Kaplan-Meier survival analysis, univariable Cox proportional hazards regression, and cardiovascular mortality assessment, have not been reported previously.

## Results

A total of 98 maintenance HD patients were enrolled and stratified into three groups according to baseline MPV: Group 1 (n=38), Group 2 (n=32), and Group 3 (n=28). As shown in Table 1, demographic and clinical characteristics were comparable across the three groups, with no statistically significant between-group differences at baseline.

**Table 1:** Baseline demographic and clinical characteristics of the study groups This table summarizes the distribution of demographic variables, comorbidities, causes of ESRD, medication use, and dialysis vintage for Group 1, Group 2, and Group 3. Data are presented as mean±SD for continuous variables and n (%) for categorical variables. P-values were obtained using ANOVA for continuous variables and the Pearson chi-squared (χ²) test for categorical variables. Statistical significance was defined as p<0.05. ESRD: end-stage renal disease; n: number; SD: standard deviation; %: percentage; χ²: chi-squared test; F: F-statistic; ANOVA: analysis of variance; mg: milligram

Characteristic	Group 1 (n=38)	Group 2 (n=32)	Group 3 (n=28)	Test statistic	P-value
Male sex, n (%)	17 (45.1)	14 (45.3)	13 (46.5)	χ²=0.000	1.000
Age (years), mean±SD	53.06±12.07	54.77±11.28	53.67±10.88	F=0.195	0.823
Diabetes mellitus, n (%)	20 (54.9)	18 (56.6)	15 (55.3)	χ²=0.132	0.869
Cardiovascular disease, n (%)	24 (62.7)	20 (64.2)	18 (64.4)	χ²=0.022	1.000
Cause of ESRD, n (%)					
Diabetic nephropathy	15 (43.1)	12 (39.6)	12 (42.9)	χ²=0.030	0.863
Hypertensive nephropathy	11 (29.4)	10 (32.1)	9 (32.3)	χ²=0.090	0.768
Chronic glomerulonephritis	6 (15.7)	4 (11.3)	4 (14.3)	χ²=0.440	0.509
Receiving antihypertensive therapy, n (%)	23 (62.7)	18 (56.6)	17 (61.1)	χ²=0.407	0.661
Aspirin (75 mg/day), n (%)	38 (100)	32 (100)	28 (100)	Not applicable	1.000
Dialysis vintage (years), mean±SD	9.38±2.09	9.45±2.01	9.41±2.07	F=0.009	0.991
Current smoker, n (%)	18 (48.1)	15 (47.2)	13 (47.8)	χ²=0.480	0.601

The proportion of male patients was similar across the groups (45.1%, 45.3%, and 46.5%, respectively; p=1.000), as was the mean age (53.06±12.07, 54.77±11.28, and 53.67±10.88 years, respectively; p=0.823).

The prevalence of diabetes mellitus did not differ significantly among the groups (54.9%, 56.6%, and 55.3%, respectively; p=0.869), nor did the prevalence of cardiovascular disease (62.7%, 64.2%, and 64.4%, respectively; p=1.000).

The distribution of the primary causes of ESRD was comparable across the three groups. Diabetic nephropathy was the most common etiology (43.1%, 39.6%, and 42.9%; p=0.863), followed by hypertensive nephropathy (29.4%, 32.1%, and 32.3%; p=0.768) and chronic glomerulonephritis (15.7%, 11.3%, and 14.3%; p=0.509).

The proportion of patients receiving antihypertensive medications was similar among the groups (62.7%, 56.6%, and 61.1%, respectively; p=0.661). All participants received a uniform antiplatelet regimen consisting of aspirin at a daily dose of 75 mg (38/38, 32/32, and 28/28 patients, respectively; 100% in each group). Dialysis vintage was also comparable (9.38±2.09, 9.45±2.01, and 9.41±2.07 years, respectively; p=0.991), as was the prevalence of current smoking (48.1%, 47.2%, and 47.8%, respectively; p=0.601).

Overall, no statistically significant between-group differences were observed in demographic characteristics, comorbidities, ESRD etiology, medication use, dialysis vintage, or smoking status. Nevertheless, because the survival analyses were unadjusted, residual confounding by measured or unmeasured factors cannot be excluded.

Laboratory parameters differed significantly among the three MPV groups (Table 2). MPV increased from 9.83±0.71 fL in Group 1 to 10.42±0.68 fL in Group 2 and 12.8 ±0.24 fL in Group 3, respectively (F(2,95)=214.84; p<0.001). In contrast, platelet count decreased progressively across the groups, from 234.02±43.10×10⁹/L to 215.13±32.18×10⁹/L and 184.65±32.10×10⁹/L (F(2,95)=14.54; p<0.001). Consequently, MPR increased significantly from 0.042±0.008 in Group 1 to 0.048±0.008 in Group 2 and 0.070±0.012 in Group 3 (F(2,95)=77.15; p<0.001).

**Table 2:** Comparison of laboratory and dialysis-related parameters among the three MPV groups Data are expressed as mean±SD. Group comparisons were performed using one-way ANOVA, with p<0.05 considered statistically significant. ANOVA: analysis of variance; ESA: erythropoiesis-stimulating agent; F: F statistic; fL: femtoliter; Hgb: hemoglobin; IU: international unit; MPV: mean platelet volume; MPR: mean platelet volume-to-platelet count ratio; n: number of patients; PLT: platelet count; URR: urea reduction ratio; g: gram; L: liter; mg: milligram

Indicator	Group 1 (n=38)	Group 2 (n=32)	Group 3 (n=28)	ANOVA, F(2,95)	P-value
MPV, fL	9.83±0.71	10.42±0.68	12.84±0.24	214.84	<0.001
PLT, ×10⁹/L	234.02±43.10	215.13±32.18	184.65±32.10	14.54	<0.001
MPR	0.042±0.008	0.048±0.008	0.070±0.012	77.15	<0.001
URR, %	74.4±3.56	73.4±2.98	63.9±2.41	107.26	<0.001
Hgb, g/L	116.12±6.74	115.15±5.32	98.95±4.21	87.78	<0.001
ESA dose, IU/week	6,142±2,080	7,225±2,150	10,718±1,853	42.39	<0.001
Albumin, g/L	44.5±3.53	39.6±3.22	32.4±2.41	119.42	<0.001

Dialysis adequacy, assessed by URR, was similar in Groups 1 and 2 but substantially lower in Group 3. The mean URR values were 74.4±3.56%, 73.4±2.98%, and 63.9±2.41%, respectively, with a significant overall difference (F(2,95)=107.26; p<0.001). Hgb concentrations were also similar in Groups 1 and 2 but markedly reduced in Group 3, with mean values of 116.12±6.74, 115.15±5.32, and 98.95±4.21 g/L, respectively (F(2,95)=87.78; p<0.001). Conversely, ESA requirements increased progressively across the groups, reaching the highest level in Group 3: 6,142±2,080, 7,225±2,150, and 10,718±1,853 IU/week, respectively (F(2,95)=42.39; p<0.001). Serum albumin decreased progressively with increasing MPV, from 44.5±3.53 g/L in Group 1 to 39.6±3.22 g/L in Group 2 and 32.4±2.41 g/L in Group 3 (F(2,95)=119.42; p<0.001).

Overall, higher MPV and MPR values were associated with lower platelet counts, poorer dialysis adequacy, lower Hgb and albumin concentrations, and higher ESA requirements. The overall ANOVA results demonstrate significant differences, but post hoc testing is required to determine which individual group pairs differ.

**Figure 1:**
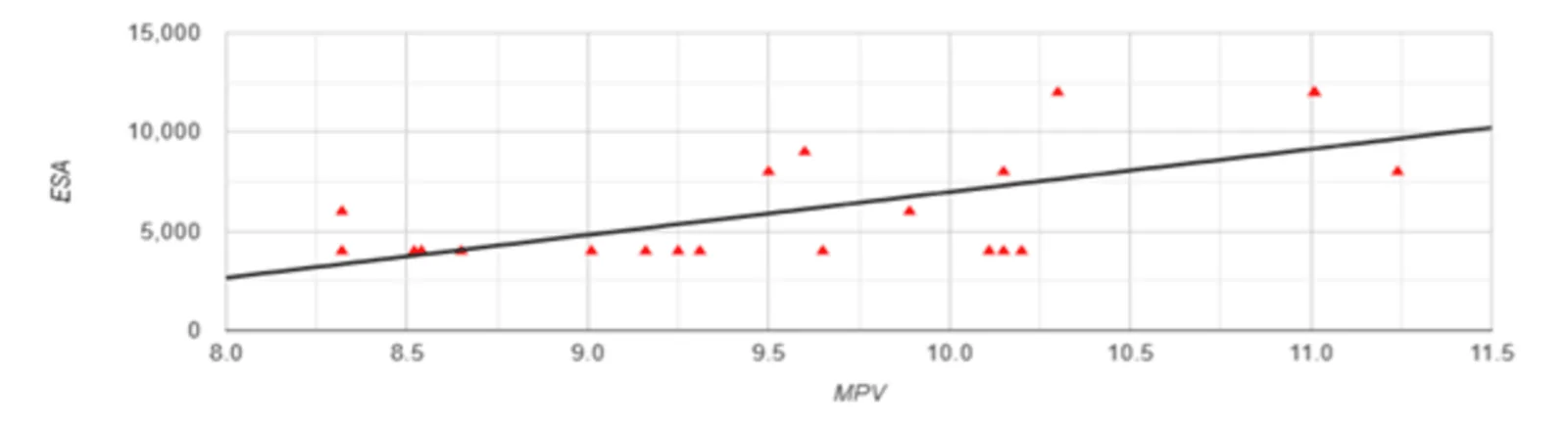
Scatter plot illustrating the relationship between MPV and weekly ESA dose Each point represents one patient, and the solid line indicates the linear regression fit. The strength of the association was assessed using Pearson's correlation coefficient (r=0.644; p=0.002). Statistical significance was defined as p<0.05. MPV: mean platelet volume; ESA: erythropoiesis-stimulating agent

Correlation analysis demonstrated a significant positive correlation between MPV and the administered ESA dose, indicating that higher MPV values were associated with increased ESA requirements (r=0.644; p=0.002) (Figure 1).

In contrast, an inverse correlation was observed between MPV and Hgb concentration; however, this association did not reach statistical significance (r=-0.363; p=0.106).

Neither serum albumin concentration nor dialysis adequacy, evaluated by URR, was significantly associated with MPV (both p>0.05).

**Figure 2:**
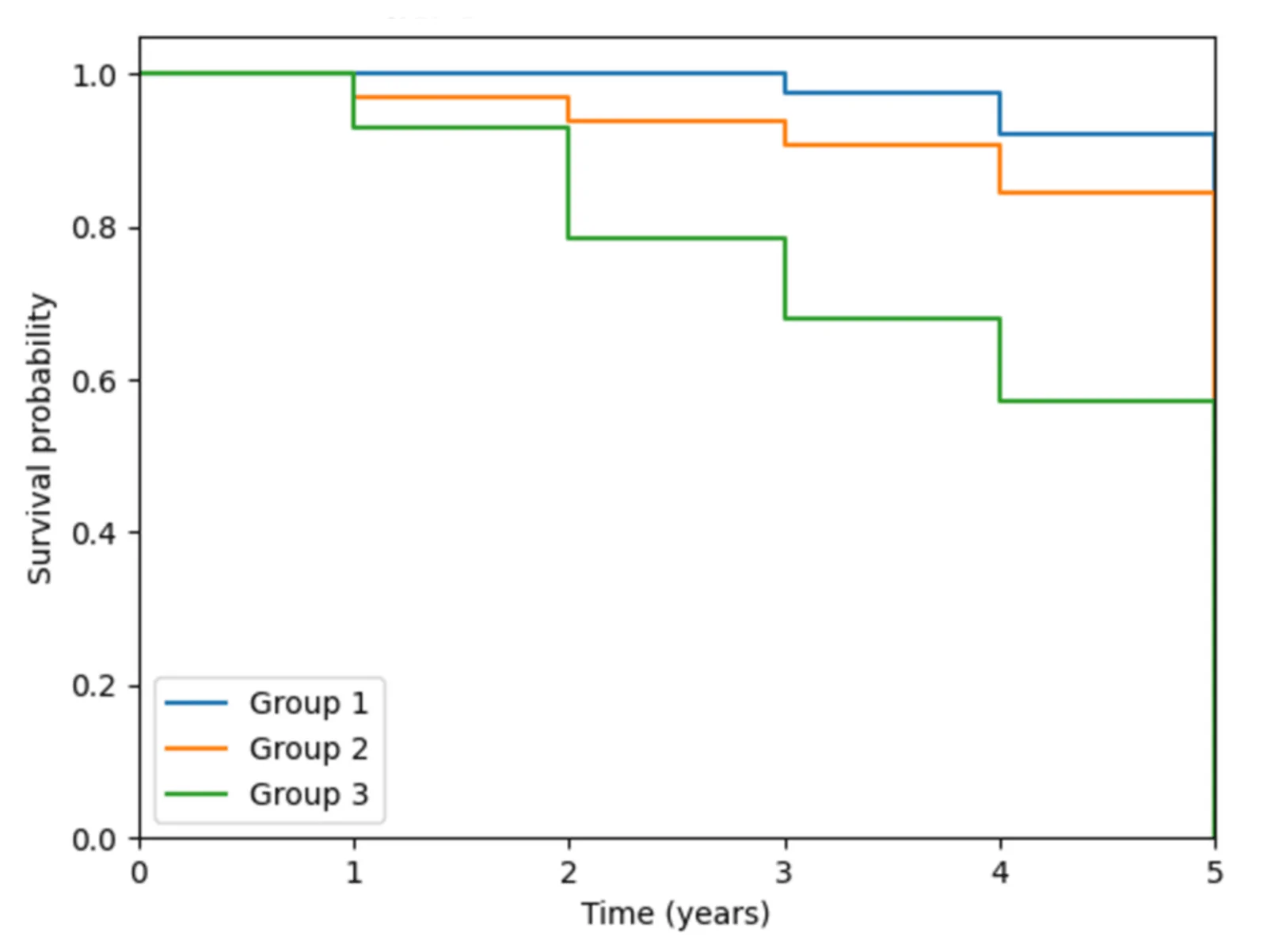
Kaplan-Meier survival curves according to baseline mean platelet volume group during the five-year follow-up period Survival probabilities are shown for Groups 1-3. Differences between survival curves were evaluated using the log-rank test (χ²=12.69; p=0.0018). Statistical significance was defined as p<0.05. blue line: expected survival for Group 1; orange line: expected survival for Group 2; green line: expected survival for Group 3

Kaplan-Meier survival analysis demonstrated a progressive decline in survival with increasing baseline MPV (Figure 2). During the five-year follow-up, five of 38 patients (13.2%) died in Group 1, compared with nine of 32 patients (28.1%) in Group 2 and 15 of 28 patients (53.6%) in Group 3, corresponding to estimated five-year survival rates of 86.8%, 71.9%, and 46.4%, respectively.

Differences in survival became evident early during follow-up. By the third year, only one death had occurred in Group 1, whereas three deaths were recorded in Group 2 and nine deaths in Group 3, corresponding to estimated three-year survival rates of 97.4%, 90.6%, and 67.9%, respectively (Table 3). The Kaplan-Meier curves separated early and remained progressively divergent throughout the observation period, with patients in the highest MPV group showing the lowest estimated survival probability.

**Table 3:** Estimated Kaplan-Meier survival probabilities at three and five years according to baseline mean platelet volume group Values are presented as percentages (%). Survival differences among groups were evaluated using the log-rank test (χ²=12.69; p=0.0018), with statistical significance defined as p<0.05.

Group	3-year survival	5-year survival
Group 1	97.4%	86.8%
Group 2	90.6%	71.9%
Group 3	67.9%	46.4%

A statistically significant difference in survival distributions was observed across the three MPV categories, as indicated by the log-rank test (χ²=12.69; p=0.0018).

To quantify the association between MPV group and mortality, a univariable Cox proportional hazards model was performed using Group 1 as the reference category (Table 4). Patients in Group 2 had a 2.28-fold higher hazard of death than those in Group 1; however, this difference was not statistically significant (HR=2.28; 95% CI 0.76-6.80; p=0.140). By comparison, patients in Group 3 had a significantly higher hazard of all-cause mortality than those in Group 1, with a 5.45-fold increase in the hazard of death (HR=5.45; 95% CI 1.98-15.02; p=0.001).

**Table 4:** Univariable Cox proportional hazards regression analysis of all-cause mortality according to baseline mean platelet volume group Hazard ratios are presented with 95% CI using Group 1 as the reference category. The estimates are unadjusted for potential confounding variables. Statistical significance was defined as p<0.05.

Variable	Hazard ratio	95% CI	P-value
Group 2 vs. Group 1	2.28	0.76-6.80	0.140
Group 3 vs. Group 1	5.45	1.98-15.02	0.001

Overall, these findings demonstrate a strong association between higher MPV and adverse long-term survival in maintenance HD patients. Patients with the highest MPV values showed a significantly higher unadjusted hazard of all-cause mortality compared with patients in the lowest MPV group, supporting the potential prognostic relevance of MPV as a readily available biomarker in this population.

To further evaluate the association of MPR with all-cause mortality, univariable Cox proportional hazards regression analysis was performed. MPR was significantly associated with all-cause mortality. Each 0.01-unit increase in MPR was associated with a 49% higher hazard of death (HR=1.49; 95% CI 1.15-2.28; p<0.001).

These findings suggest that higher MPR values are associated with a higher hazard of mortality. Given that MPR integrates both platelet size and platelet count, it may provide additional information regarding platelet activation and thrombo-inflammatory status, highlighting its potential prognostic relevance as an inexpensive and readily available hematological index in maintenance HD patients. However, because the Cox analyses were unadjusted, these associations should not be interpreted as demonstrating independent prognostic effects.

**Figure 3:**
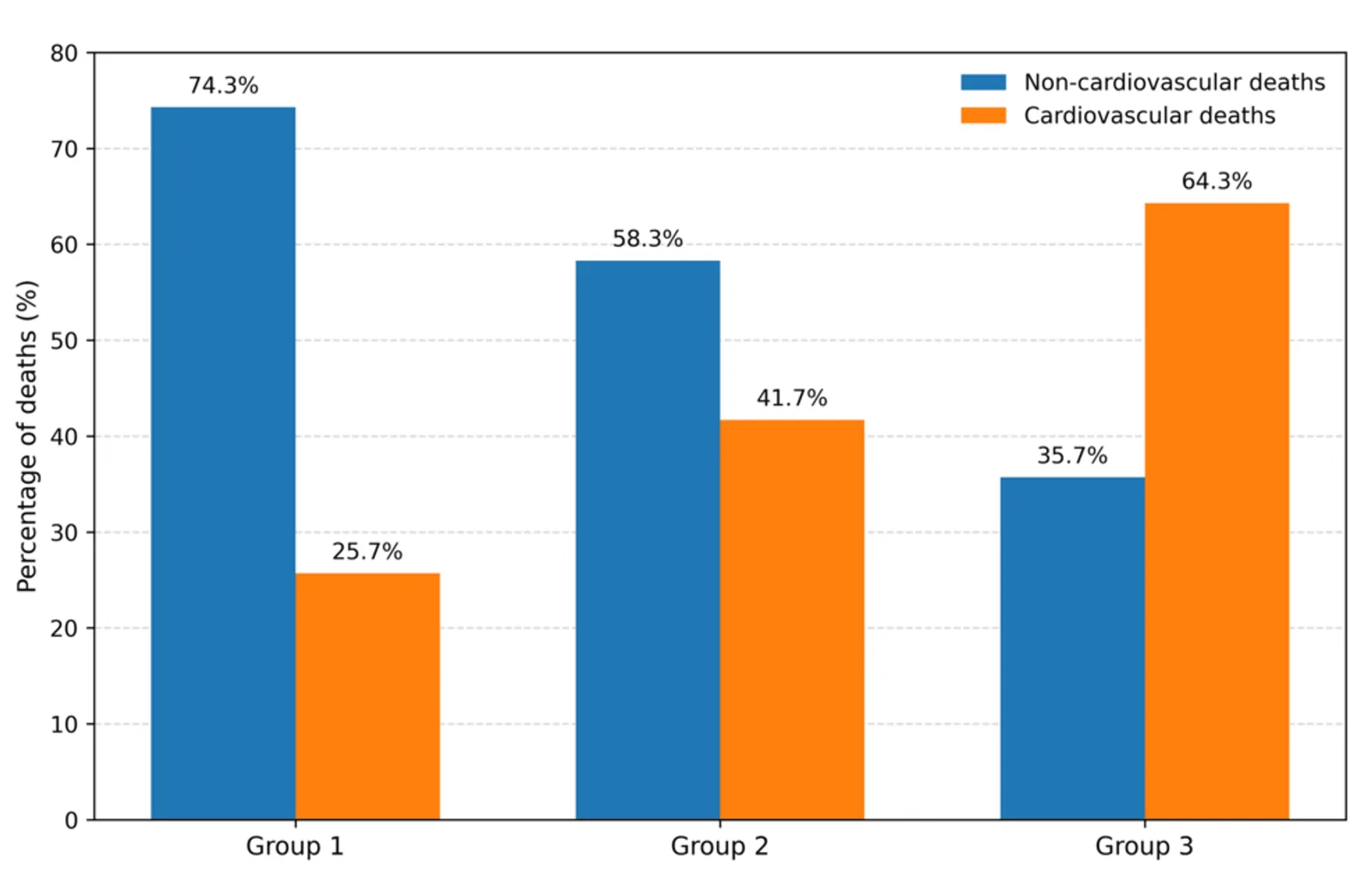
Distribution of cardiovascular and non-cardiovascular deaths according to baseline mean platelet volume group Bars represent the percentage (%) of deaths within each mean platelet volume group. Differences among groups were analyzed using the Pearson chi-squared (χ²) test (χ²=11.30; p=0.0035). Statistical significance was defined as p<0.05. blue column: non-cardiovascular deaths; orange column: cardiovascular deaths

Analysis of the causes of death demonstrated a progressive increase in cardiovascular mortality with increasing MPV (Figure 3). Cardiovascular death was progressively more frequent with increasing baseline MPV, occurring in 26.3% of patients in Group 1, 43.8% in Group 2, and 67.9% in Group 3. Pearson's chi-squared analysis confirmed a significant association between MPV group and cardiovascular mortality (χ²=11.30; p=0.0035).

Compared with patients in the lowest MPV group (Group 1), those in the highest MPV group (Group 3) had a 2.58-fold higher relative risk of cardiovascular death (RR=2.58; 95% CI 1.43-4.65). Compared with the intermediate MPV group (Group 2), patients in Group 3 had a 1.55-fold higher relative risk of cardiovascular death (RR=1.55; 95% CI 0.97-2.48). Although the risk of cardiovascular mortality was significantly higher in Group 3 than in Group 1, the difference between Groups 3 and 2 was not statistically significant.

## Discussion

The results of this study identify MPV and MPR as important indicators associated with adverse clinical outcomes among maintenance HD patients. Patients with higher MPV values exhibited significantly lower platelet counts, reduced dialysis adequacy, lower Hgb and serum albumin concentrations, greater ESA requirements, poorer long-term survival, and a substantially higher frequency of cardiovascular death. These findings support the hypothesis that increased platelet activation represents an important component of the complex pathophysiology underlying cardiovascular disease in patients with ESRD.

One of the principal findings of our study is the progressive deterioration in clinical and laboratory parameters with increasing MPV. Higher MPV was accompanied by lower platelet counts and significantly higher MPR values, suggesting enhanced platelet activation and increased platelet consumption. These findings extend previous observations [7,11] by demonstrating that elevated MPV is associated not only with platelet activation but also with poorer dialysis adequacy, greater anemia severity, and reduced long-term survival, reinforcing its potential role as an integrated marker of adverse clinical status in maintenance HD patients.

Another important observation was the significant association between elevated MPV and markers of inadequate dialysis and poorer nutritional status. One possible explanation is that inadequate dialysis promotes the retention of uremic toxins, oxidative stress, and persistent systemic inflammation, all of which have been implicated in platelet activation and alterations in platelet morphology [5,6]. Patients with the highest MPV demonstrated lower mean URR and serum albumin values. Similar associations between impaired nutritional status, chronic inflammation, and adverse outcomes have been consistently reported in maintenance HD patients, with serum albumin recognized as one of the strongest predictors of mortality [14]. Although linear correlations between MPV and URR or serum albumin were not statistically significant in our cohort, the marked differences across MPV categories suggest that these relationships may be more complex than simple linear associations. The coexistence of elevated MPV, lower albumin, and reduced dialysis adequacy therefore supports the concept that platelet activation may reflect an overall adverse inflammatory and metabolic profile rather than an isolated hematological abnormality.

Our study also identified a strong positive correlation between MPV and weekly ESA dose. Patients with higher MPV received greater doses of ESA and had lower mean Hgb concentrations, suggesting a possible relationship between platelet activation and ESA hyporesponsiveness. Although direct evidence linking MPV with ESA responsiveness remains limited, chronic inflammation is a well-established determinant of ESA resistance in maintenance HD patients [15,16]. Inflammatory cytokines impair erythropoiesis, promote functional iron deficiency, and may also contribute to alterations in platelet production and morphology. Therefore, elevated MPV may indirectly reflect the inflammatory milieu associated with impaired erythropoietic response rather than platelet activation alone.

Perhaps the most clinically relevant finding was the marked difference in long-term survival among the three MPV groups. Kaplan-Meier analysis demonstrated early separation of the survival curves, with patients in the highest MPV group experiencing the poorest five-year survival. Univariable Cox proportional hazards analysis further demonstrated that patients with MPV >11.0 fL had more than a fivefold higher hazard of death compared with those with MPV <10.0 fL. These observations indicate that elevated MPV is associated with adverse prognosis and may have potential prognostic relevance in maintenance HD patients. However, because the Cox analysis was unadjusted, this association should not be interpreted as demonstrating an independent prognostic effect.

The present results are in agreement with earlier evidence that platelet size is associated with adverse outcomes in high-risk populations. In maintenance HD, increased MPV may represent the hematological expression of several interrelated processes, including persistent inflammation, oxidative injury, and enhanced platelet activation. Its routine availability as part of the complete blood count may therefore make MPV a potentially useful marker for further prognostic investigation [17,18]. Evidence from a large national cohort of incident HD patients further supports this interpretation. Kim et al. identified a graded relationship between MPV and all-cause mortality, with the risk increasing across higher MPV categories in analyses based on both initial and repeatedly updated measurements [19].

MPR appeared to provide potentially complementary prognostic information to MPV alone. Because this index incorporates both platelet size and platelet count, it may provide additional information regarding platelet activation and thrombo-inflammatory status. In the present cohort, increasing MPR was significantly associated with a higher hazard of all-cause mortality in univariable Cox regression, with each 0.01-unit increment corresponding to a 49% increase in the hazard of death. Given that MPR is derived from routinely available complete blood count parameters without additional cost, these findings support its potential prognostic relevance in maintenance HD. However, its independent prognostic value and incremental predictive performance beyond established risk factors could not be determined in the present study. Our results are in agreement with previous reports demonstrating the prognostic significance of MPR in cardiovascular disease and other chronic inflammatory conditions [13,20,21].

Collectively, these observations suggest that MPR may represent a potentially informative marker of the thrombo-inflammatory state associated with chronic kidney disease and warrant further investigation alongside MPV.

The analysis of cardiovascular mortality also demonstrated an association between higher MPV categories and cardiovascular death. Cardiovascular deaths increased progressively across MPV categories, and patients with MPV >11.0 fL exhibited more than a twofold higher relative risk of cardiovascular death than those with MPV <10.0 fL. The present findings are consistent with previous evidence identifying elevated MPV as a marker of increased CVR and mortality in chronic kidney disease, as well as in other populations at high CVR [8,9]. This concept is further supported by a multicenter study of 518 maintenance HD patients, in which coronary heart disease was significantly more prevalent among individuals in the highest MPV quintile than among those in the lowest quintile, independent of demographic characteristics, traditional CVR factors, and laboratory variables [22]. Although causality cannot be inferred from a retrospective observational study, our findings further support the hypothesis that enhanced platelet activation, reflected by elevated MPV, may be associated with cardiovascular complications in patients undergoing maintenance HD [10,23].

The present findings may warrant further investigation regarding their potential implications for routine nephrology practice. Because MPV and MPR are obtained from the complete blood count without additional costs or specialized testing, they may represent accessible candidate markers for future risk stratification studies in patients undergoing maintenance HD. Whether MPV and MPR provide independent or incremental prognostic information beyond established clinical risk factors should be addressed in future prospective multicenter studies using appropriately adjusted multivariable models and external validation.

Strengths and limitations

The present investigation benefits from several methodological strengths that should be considered when interpreting the findings. First, all patients were managed according to a standardized HD protocol, including identical dialysis schedules, uniform dialysis equipment, standardized anticoagulation with low-molecular-weight heparin, a consistent antiplatelet regimen, and comparable baseline ESA dosing. This standardized clinical approach minimized treatment-related variability and improved the comparability of treatment exposure across the study population. Second, laboratory analyses were performed using validated automated methods under routine quality-controlled conditions, with hematological parameters obtained exclusively from pre-dialysis blood samples to avoid the influence of ultrafiltration-induced hemoconcentration. Third, the use of annual mean values derived from repeated laboratory measurements reduced the impact of short-term biological fluctuations and provided a more representative assessment of each patient's long-term clinical status. In addition, the study evaluated both MPV and MPR, together with dialysis adequacy, anemia-related parameters, nutritional status, survival, and cardiovascular mortality, providing a comprehensive assessment of their associations with adverse clinical outcomes. Finally, complete clinical and laboratory data were available for all included patients, eliminating the need for data imputation and minimizing potential bias arising from missing data.

Several limitations should also be acknowledged. The retrospective, single-center nature of the study precludes conclusions regarding causality and may limit the applicability of the findings to broader maintenance HD populations. Although the influence of some potential confounding factors was reduced through strict eligibility criteria and standardized treatment protocols, residual confounding from measured and unmeasured variables cannot be completely excluded. Importantly, the Cox proportional hazards analyses were univariable and were not adjusted for established mortality risk factors or other potential confounders. Consequently, the reported HRs should be interpreted as unadjusted associations and do not establish MPV or MPR as independent prognostic factors. Unmeasured variables, including inflammatory biomarkers (e.g., C-reactive protein, interleukin-6), nutritional indices, and dialysis membrane biocompatibility, may also have influenced the observed associations.

The relatively small sample size and limited number of mortality events restricted the statistical power of subgroup analyses and the ability to construct robust multivariable prognostic models without a substantial risk of overfitting. This limitation is particularly relevant to the analysis of cardiovascular mortality. Furthermore, MPV measurements may vary according to the hematology analyzer used and the interval between blood collection and analysis, although standardized laboratory procedures were applied consistently throughout the study to minimize analytical variability. The proportional hazards assumption was not formally reassessed in the present secondary analysis, which represents an additional limitation of the Cox regression findings. The homogeneous, single-center nature of the study population may restrict the wider applicability of the results. In addition, no external validation or formal assessment of incremental predictive performance was performed; therefore, the clinical utility of MPV and MPR for risk prediction cannot be established from the present study. Replication in larger, geographically diverse, prospective multicenter cohorts using appropriately adjusted multivariable models and external validation will be essential to confirm the robustness and generalizability of these findings.

Overall, despite these limitations, our findings suggest that MPV and MPR are associated with adverse clinical outcomes and may represent readily available candidate markers for further prognostic investigation in patients receiving maintenance HD.

## Conclusions

In this retrospective cohort of patients receiving maintenance HD, elevated MPV and MPR were associated with impaired dialysis adequacy, higher ESA requirements, lower Hgb and serum albumin concentrations, increased cardiovascular mortality, and reduced long-term survival. Patients with the highest MPV values demonstrated significantly worse clinical outcomes and a markedly increased unadjusted hazard of all-cause mortality. These findings suggest that routinely available platelet indices, including MPV and MPR, may have potential prognostic relevance in maintenance HD patients; however, their independent predictive value and clinical utility remain to be established. Prospective multicenter studies incorporating appropriately adjusted multivariable analyses and external validation are required to determine whether these markers improve prognostic models and can contribute to individualized patient management.
